# Impact of Zirconia Coping Overlapped by Lithium Disilicate on Esthetic Outcome: A Systematic Review

**DOI:** 10.7759/cureus.100509

**Published:** 2025-12-31

**Authors:** Mousa Alrashidy, Raghdah Abdullah Al Thubaiti, Najla Haif Alqahtani, Abdulrahman Kamal Habash, Ruba Abdullah Alkhalil

**Affiliations:** 1 Restorative Dentistry, Prince Abdulrahman Advanced Dental Institute, Riyadh, SAU; 2 Dentistry, Prince Abdulrahman Advanced Dental Institute, Riyadh, SAU; 3 Dentistry, Ministry of Health, Riyadh, SAU; 4 Dentistry, Ministry of Health, Qassim, SAU

**Keywords:** bilayered restoration, dental aesthetics, esthetic outcome, lithium disilicate, systematic review, zirconia, zirconia-reinforced lithium silicate (zls)

## Abstract

This systematic review aims to evaluate the impact of a zirconia coping veneered or overlapped by lithium disilicate ceramic on the esthetic and mechanical outcomes of dental restorations. A systematic search of PubMed, Medline, Embase, and the Cochrane Library was conducted for articles published between January 2014 and January 2023. Inclusion criteria encompassed primary studies investigating the aesthetic outcome of restorations with a zirconia coping layered with lithium disilicate. The review included 13 studies (10 in vitro, three clinical) after screening, data extraction, and risk of bias assessment. The findings indicate a predominantly favorable consensus, with 10 studies supporting the bilayered approach. This combination leverages the high strength of zirconia and the superior aesthetics of lithium disilicate, demonstrating excellent fracture resistance, clinical success rates, and patient satisfaction in the short to medium term. Key factors influencing aesthetics include the ability to polish zirconia-reinforced lithium silicate (ZLS) to a high gloss and the critical role of cement shade on the final color. However, some studies reported less favorable outcomes, including higher plaque accumulation compared to monolithic zirconia and potentially higher flexural strength for traditional lithium disilicate over some ZLS materials. The zirconia coping and lithium disilicate veneer combination is a viable and aesthetically promising treatment modality. It offers a favorable balance of strength and aesthetics, though the results are technique-sensitive and influenced by material processing. The promising in-vitro and short-term clinical data necessitate confirmation through well-designed, long-term clinical trials to definitively establish the clinical longevity and aesthetic stability of these restorations.

## Introduction and background

The success of artificial teeth depends not only on their dimensions, texture, and contours but also on their ability to replicate the light behavior of natural dentition. Key optical properties such as translucency, metamerism, and opalescence are critical in this regard [1]. Dental ceramics, particularly zirconia-based and lithium disilicate crowns, exhibit significant differences in their translucency. While various grades of translucent zirconia are available, its higher crystalline content generally renders it less translucent compared to lithium disilicate [2].

Among the most prevalent materials for single crowns are lithium disilicate and monolithic zirconia, prized for their aesthetic outcomes that closely mimic natural teeth [3]. These ceramics are highly aesthetic, biocompatible, and resistant. However, their inherent hardness and brittleness can lead to crack propagation under load and wear of the antagonistic teeth [4]. Zirconium dioxide (zirconia) has gained widespread popularity in modern dentistry due to its high biocompatibility [5,6], low plaque adhesion [7], high flexural strength and toughness [8], absence of mucosal discoloration [9], and favorable aesthetic properties [10].

Lithium disilicate and monolithic zirconia are both offered in CAD/CAM blocks and discs with different translucency levels and hues. Because of its translucency, lithium disilicate has a distinctive crystal microstructure, great strength, and remarkable aesthetics [11]. After a final crystallization firing, this material, which is milled in a partially crystallized form, reaches its maximum strength and aesthetic potential [12]. Despite the introduction of high-translucency zirconia for monolithic restorations, it remains comparatively opaque [13,14], limiting its use as a monolithic material primarily to the posterior region [5,6]. Conversely, veneered zirconia restorations have been associated with a significant clinical incidence of chipping and delamination of the veneering ceramic [15], with framework fracture being a less common complication [10,16].

Newer materials, such as zirconia-reinforced lithium silicate (ZLS), have been created to overcome these difficulties. ZLS is based on a lithium-metasilicate glass ceramic supplemented with roughly 10% zirconium dioxide, generating a fine-grained microstructure following crystallization [17]. This new generation of CAD/CAM material aspires to combine the positive mechanical qualities of zirconia with the superior visual look of glass-ceramics [17]. Longer-term data are required, although preliminary research suggests that ZLS restorations have a high clinical success rate after a year [18]. Monolithic zirconia and lithium disilicate pressed on zirconia frameworks exhibit better fracture resistance than monolithic lithium disilicate while still providing similar aesthetics, according to in-vitro experiments [19]. Furthermore, zirconia-reinforced lithium silicate glass-ceramics (ZLS) are considered a biocompatible material with fracture resistance adequate for physiological chewing loads [20,21].

In pursuit of better aesthetics, the manufacturing process of monolithic zirconia has been refined by increasing the cubic zirconia phase, reducing aluminum content and grain size, and increasing density. These changes enhance translucency and reduce light scattering, albeit at the cost of some material strength [22,23]. ZLS glass ceramics, with a flexural strength ranging from 370 to 420 MPa, are now widely used as machinable ceramics for CAD/CAM techniques, offering strength comparable to lithium disilicate and a better aesthetic result and bond strength than zirconia ceramics [24]. The performance of both zirconia and ZLS ceramics with minimally invasive vertical preparation has been found to be comparable to that of conventional preparations [25].

A defining characteristic of ZLS is its crystalline structure, which contributes to its translucency [4,26]. The combination of a glassy phase and translucent crystals allows it to meet high aesthetic expectations [27]. However, the translucency of any ceramic material, including ZLS, presents a challenge in color-matching due to the influence of light reflection from the underlying tooth structure or coping [27]. This systematic review aims to evaluate and synthesize the available evidence on the impact of a zirconia coping overlapped or veneered by lithium disilicate ceramic on the overall esthetic outcome of dental restorations.

## Review

Methods

Search Strategy

This systematic review was conducted by searching the electronic databases PubMed, Medline, Embase, and the Cochrane Library. A comprehensive search strategy was developed using key terms related to "zirconia," "lithium disilicate," "coping," "overlap," "aesthetic outcome," and their synonyms to identify relevant studies. The search was limited to articles published in English between January 2014 and January 2023. All retrieved articles were screened by their titles and abstracts to identify those that specifically addressed the effect of a zirconia coping veneered or overlapped by lithium disilicate on aesthetic outcomes.

Inclusion and Exclusion Criteria

In consultation with a dental expert, the inclusion criteria were defined as all primary research studies published in English between January 2014 and January 2023 that directly investigated the aesthetic outcome of restorations involving a zirconia coping layered with lithium disilicate ceramic. The exclusion criteria disqualified all review articles (narrative reviews, systematic reviews, and meta-analyses), duplicate publications, commentaries, and studies that did not measure or report on aesthetic outcomes relevant to the prosthesis.

Study Selection and Data Extraction

A full-text review of possibly pertinent papers was conducted after titles and abstracts were screened against the eligibility criteria. One researcher used a standardized form to obtain data from the final included studies. Authors, year of publication, sample size, study design (e.g., in-vitro, clinical trial), aesthetics assessment methodology, important aesthetics-related findings, and the study's conclusion were among the extracted data. The accuracy of the extracted data was independently examined by another investigator, and any disagreements were settled through conversation.

Assessment of Risk of Bias

Two investigators independently assessed the methodological quality and risk of bias for each of the included studies. A pre-specified set of criteria tailored to specific study designs (e.g., using tools like Cochrane RoB for clinical trials or modified checklists for in-vitro studies) was used for this assessment [21]. Studies that were judged to have a high risk of bias were excluded from the final synthesis to ensure the robustness of the review's findings.

Results

**Figure 1:**
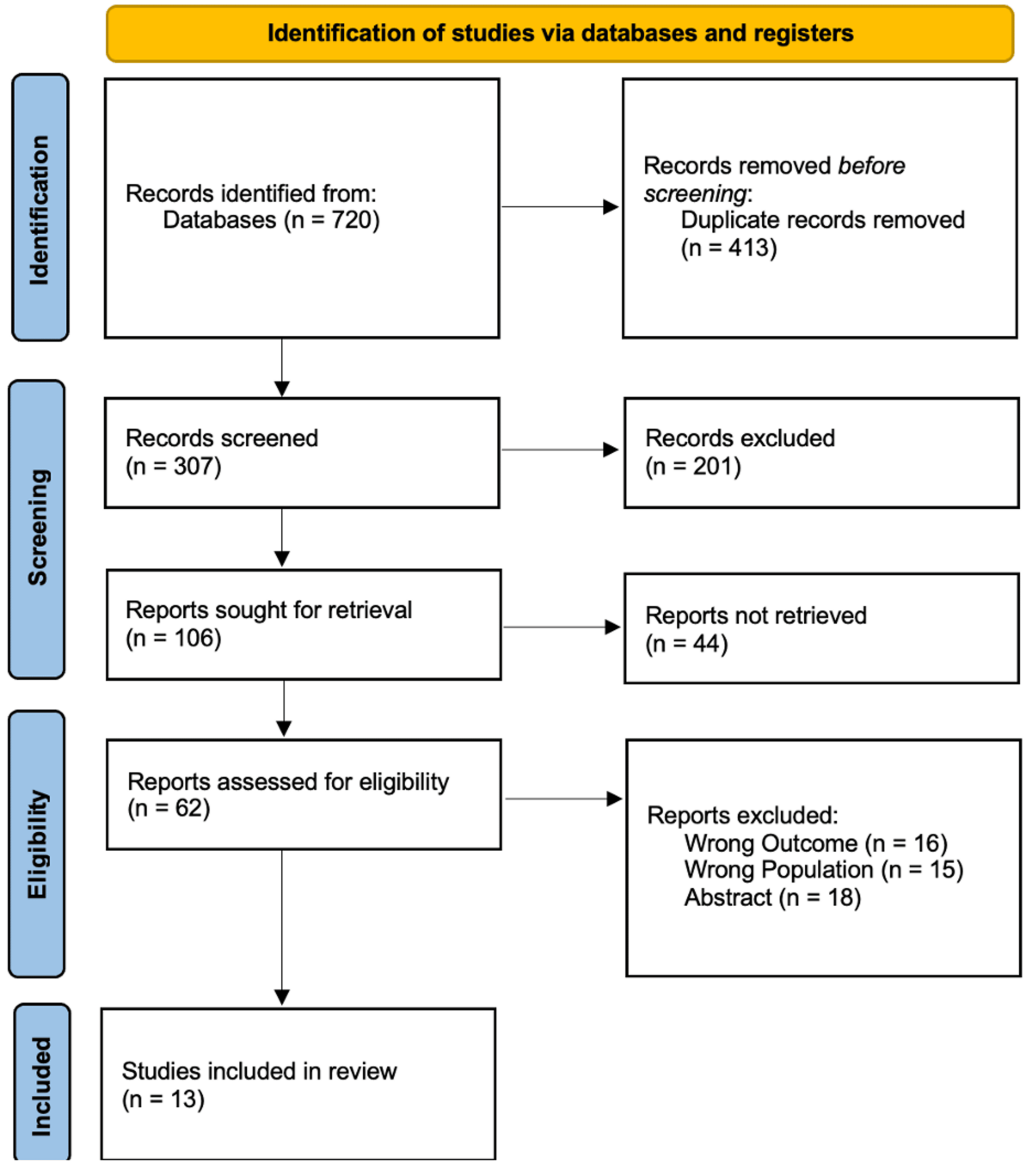
PRISMA Flow Diagram Illustrating Study Selection Process

The PRISMA flow diagram in Figure 1 outlines the systematic study selection process. Initially, 720 records were identified through database searches. After removing 413 duplicates, 307 titles and abstracts were screened, leading to 106 reports sought for retrieval. Following a full-text assessment of 62 articles for eligibility, 49 were excluded due to wrong outcomes, wrong population, or being only abstract, resulting in 13 studies being included in the final systematic review.

Tables 1, 2 illustrates that the systematic review included a total of 13 studies [18,19,28-38], comprising ten in-vitro studies and three prospective clinical follow-up studies [18,19,24,28-33,35-38]. The overall findings indicated that 10 of the included studies reported favorable outcomes for restorations involving a zirconia coping layered with lithium disilicate, particularly concerning fracture resistance and clinical performance. However, three studies drew conclusions that were less favorable or highlighted advantages of other materials, primarily relating to mechanical properties and periodontal responses [33,34,37].

**Table 1:** Summary of Included Studies

Author (Year)	Study Design	Sample Size / Model	Key Intervention / Comparison	Primary Outcome Measure(s)
Pozzi et al., 2015 [28]	Prospective Clinical	16 patients	Monolithic LD crowns on CAD/CAM zirconia bridges	Survival rate, success rate, complications
Traini et al., 2016 [29]	In-vitro	Not Specified	ZLS (Partially vs. Fully Crystallized)	Fracture Toughness (Ft), Vickers Hardness (HV)
Zimmermann et al., 2017 [18]	Prospective Clinical	60 restorations	ZLS CAD/CAM Restorations	Clinical success rate, fracture rate
Choi et al., 2017 [19]	In-vitro	3-unit FDP model	LD pressed on Zr vs. Monolithic LD vs. Monolithic Zr	Fracture Resistance, Failure Mode
Fathy et al., 2018 [30]	In-vitro	25 specimens	ZLS aged in different pH media	Surface Roughness (Ra), Wear against Enamel
Riccitiello et al., 2018 [31]	In-vitro	Not Specified	CAD-CAM Zr vs. CAD-CAM LD vs. Heat-Pressed LD	Marginal and Internal Adaptation
Vichi et al., 2018 [32]	In-vitro	Not Specified	ZLS (Suprinity) vs. LD (e.max CAD)	Surface Roughness, Gloss after Polishing
Roh et al., 2019 [33]	Prospective Clinical	17 patients (60 prostheses)	LD pressed on Zr vs. Monolithic Zr	Periodontal Health, Bone Resorption, Complications
Ko et al., 2020 [34]	In-vitro	Not Specified	Zirconia-LS2 Bilayered Crowns vs. PFM	Fracture Resistance
Yildirim et al., 2021 [35]	In-vitro	Not Specified	ZLS vs. LD with different cement shades	Translucency, Final Color (ΔE)
Attia et al., 2021 [36]	In-vitro	Not Specified	Various Monolithic Ceramics vs. Zirconia Antagonist	Wear, Surface Roughness
Corado et al., 2022 [37]	In-vitro	Not Specified	LD Glass-Ceramics vs. ZLS	Flexural Strength
Dolve et al., 2023 [38]	In-vitro	Not Specified	Monolithic Zr vs. LD after Staining	Light Reflection Percentage

**Table 2:** Key Findings and Conclusions on Aesthetic and Mechanical Outcomes

Author (Year)	Primary Findings Related to Zirconia/LD	Conclusion Regarding Zirconia Coping with Lithium Disilicate
Pozzi et al., 2015 [28]	100% prosthetic success; high patient satisfaction; one minor ceramic chip.	Favorable. A clinically viable and successful medium-term option for full-arch rehabilitation.
Traini et al., 2016 [29]	Fully crystallized ZLS is tougher and harder; partially crystallized state is brittle.	Favorable (with processing). Final crystallization is critical for achieving optimal mechanical properties.
Zimmermann et al., 2017 [18]	96.7% success rate; two bulk fractures.	Cautiously Favorable. High short-term success, but long-term durability requires further evaluation.
Choi et al., 2017 [19]	LD pressed on Zr had superior fracture resistance to monolithic LD.	Favorable. The bilayered approach offers a strong, reliable alternative for fixed dental prostheses.
Fathy et al., 2018 [30]	Acidic pH increases roughness; causes minimal wear to enamel.	Context-dependent. Aesthetically stable in terms of enamel wear, but chemical environment affects surface.
Riccitiello et al., 2018 [31]	CAD-CAM zirconia copings had the best marginal fit.	Favorable for zirconia framework. Supports the precision of zirconia copings as a foundation.
Vichi et al., 2018 [32]	ZLS (Suprinity) can achieve high gloss and low roughness with proper polishing.	Favorable. ZLS responds well to finishing, which is crucial for final aesthetic appearance.
Roh et al., 2019 [33]	Higher plaque indices with LD-pressed Zr; lower bone resorption.	Less Favorable. The design may promote periodontal inflammation, offsetting some clinical benefits.
Ko et al., 2020 [34]	No significant difference in fracture resistance based on zirconia core design.	Favorable. The bilayered crown design itself is robust for anterior teeth.
Yildirim et al., 2021 [35]	ZLS and LD have similar translucency; cement choice is critical for color.	Neutral. The aesthetic potential is equivalent to LD, but technique-sensitive due to cement influence.
Attia et al., 2021 [36]	Zirconia antagonist causes abrasive wear to all ceramics, increasing roughness.	Unfavorable for antagonist. Using zirconia against the bilayered restoration may compromise its surface.
Corado et al., 2022 [37]	Lithium disilicate had higher flexural strength than ZLS.	Less Favorable. Pure lithium disilicate may be mechanically superior to zirconia-reinforced lithium silicate.
Dolve et al., 2023 [38]	Monolithic zirconia has higher light reflection than LD, even after staining.	Less Favorable for aesthetics. The higher reflectivity may make monolithic Zr less vital-looking than LD.

Pozzi et al. (2015) [28] conducted a prospective study assessing monolithic lithium disilicate crowns bonded to CAD/CAM zirconia complete-arch implant bridges over 3-5 years. They reported 100% survival and success rates, with only one minor veneering chip incident, concluding that this approach yielded favorable medium-term results. Similarly, Zimmermann et al. (2017) [18] found zirconia-reinforced lithium silicate (ZLS) restorations had a 96.7% clinical success rate after 12 months, despite two bulk fractures. In contrast, Roh et al. (2019) [33] reported that while implant survival was 100%, lithium disilicate-pressed zirconia prostheses showed significantly higher plaque accumulation and periodontal inflammation indices compared to monolithic zirconia, though they exhibited less bone resorption.

In-vitro studies provided insights into the material properties. Traini et al. (2016) [29] demonstrated that fully crystallized ZLS had significantly improved fracture toughness and hardness compared to its partially crystallized state. Choi et al. (2017) [19] found that lithium disilicate pressed on zirconia (LZ) frameworks had significantly higher fracture resistance than monolithic lithium disilicate (ML) and was comparable to monolithic zirconia (MZ). Studies by Fathy et al., 2018 [30] and Attia et al., 2021 [36] focused on surface interactions, showing that ZLS ceramics caused minimal wear to opposing enamel but were susceptible to increased roughness in acidic environments and when opposed by zirconia.

Research by Riccitiello et al. (2018) [31] and Vichi et al. (2018) [32] highlighted the importance of manufacturing and finishing. CAD/CAM zirconia copings showed the best marginal fit, while proper polishing of ZLS ceramics was crucial for achieving low roughness and high gloss. Aesthetic studies by Yildirim et al. (2021) [35] found that ZLS and lithium disilicate had similar translucency, with the cement color being a more critical factor for final color than the material itself. However, not all findings favored zirconia-based bilayered systems. Corado et al. (2022) [37] concluded that traditional lithium disilicate glass-ceramics possessed higher flexural strength than ZLS materials. Dolve et al. (2023) [38] reported that monolithic zirconia had higher light reflectivity than lithium disilicate, even after staining, which could impact aesthetic vitality.

Table 3 shows the risk of bias assessment reveals significant concerns, primarily due to a consistent lack of blinding for operators and/or assessors across all studies.

**Table 3:** Risk of Bias Assessment of Included Studies

Study (Author, Year)	Study Design	Domain 1: Sample Specification & Preparation	Domain 2: Blinding of Operator/Assessor	Domain 3: Randomization & Allocation	Domain 4: Incomplete Data Reporting	Domain 5: Selective Reporting	Overall Risk of Bias
Pozzi et al., 2015 [28]	Prospective	+	±	-	+	+	Some Concerns
Traini et al., 2016 [29]	In-vitro	±	-	±	+	+	High
Zimmermann et al., 2017 [18]	Prospective	+	-	-	+	+	High
Choi et al., 2017 [19]	In-vitro	+	-	+	+	+	Some Concerns
Fathy et al., 2018 [30]	In-vitro	+	-	+	+	+	Some Concerns
Riccitiello et al., 2018 [31]	In-vitro	±	-	N/A	+	+	High
Vichi et al., 2018 [32]	In-vitro	±	-	N/A	+	+	High
Roh et al., 2019 [33]	Prospective	+	-	-	+	+	High
Ko et al., 2020 [34]	In-vitro	±	-	+	+	+	Some Concerns
Yildirim et al., 2021 [35]	In-vitro	+	-	+	+	+	Some Concerns
Attia et al., 2021 [36]	In-vitro	+	-	+	+	+	Some Concerns
Corado et al., 2022 [37]	In-vitro	+	-	+	+	+	Some Concerns
Dolve et al., 2023 [38]	In-vitro	+	-	+	+	+	Some Concerns

Discussion

This systematic review aimed to synthesize the available evidence on the impact of a zirconia coping veneered with lithium disilicate on the aesthetic outcome of dental restorations. The findings from the included studies indicate a generally favorable consensus, with 10 out of the 13 reviewed studies supporting the use of this bilayered approach for aesthetic purposes [18,19,28-32,35,36,38].

The favorable clinical performance of zirconia-based frameworks is well-documented. Supporting this, Papaspyridakos and Lal [39] affirmed the viability of CAD/CAM zirconia implant-supported fixed partial dentures (FPDs) over 2-4 years, while Larsson et al. [40] reported no framework fractures in zirconia-based complete-arch restorations after three years, alongside high patient satisfaction. This long-term success is attributed to zirconia's high strength, biocompatibility, low plaque adhesion, and excellent aesthetic properties, which promote stable soft tissue integration [39-41]. To maximize these benefits, precise implant placement and minimal post-sintering adjustments of the zirconia framework are crucial to prevent surface microcracks that could compromise integrity [28].

Zirconia-reinforced lithium silicate (ZLS) is an important development in material science. According to Traini et al. [29], fully crystallized ZLS can bear posterior masticatory stresses because it has mechanical qualities that are better than those of conventional lithium disilicate and comparable to some zirconia ceramics [42]. Although it is interesting that certain studies have identified standard lithium disilicate (e.max CAD) to offer better biaxial strength than certain ZLS materials (Celtra Duo) [43], the increased strength is attributable to the insertion of zirconia crystals into the lithium silicate matrix [42]. The crystallization process is critical, as the partially crystallized state of ZLS is brittle and requires careful handling during laboratory procedures to avoid cracks [29]. The resulting fine-grained microstructure after crystallization is responsible for the improved mechanical properties, even if the individual zirconia particles are not distinctly identifiable [29,44].

Aesthetic outcomes are influenced by both material properties and technical procedures. The ability to enhance aesthetics through external staining and glazing is a key advantage, as demonstrated by Dolve et al. [38], who found that glazing reduced light reflectivity, thereby improving the vitality of the restoration. This finding is consistent with other research indicating that glazing can increase the perceived translucency of zirconia [45]. Furthermore, the wear characteristics of these materials are clinically relevant. Attia et al. [36] reported that ZLS exhibited lower wear against enamel compared to lithium disilicate, which may be due to ceramic strengthening mechanisms that resist abrasive wear [46], despite its lower fracture toughness and surface hardness compared to zirconia [36,47].

The structural integrity of the bilayered system was a focus of several studies. Ko et al. [34] found that the fracture resistance of zirconia-lithium disilicate bilayered crowns was not significantly affected by the coping design, suggesting robust performance. The strong bond at the interface, potentially reinforced by an ion-migration reaction layer, is thought to compensate for the differences in mechanical properties between the high-strength zirconia core and the more aesthetic lithium disilicate veneer [48-52]. However, chipping of the veneering ceramic remains a documented complication in zirconia-based restorations on both teeth [53, 54] and implants [55], underscoring the importance of optimal framework design, processing, and handling to minimize this risk [55-57].

Despite the overall positive findings, three studies presented less favorable or comparative outcomes [33,34,37]. Roh et al. [33] reported higher plaque indices and periodontal inflammation around lithium disilicate-pressed zirconia prostheses compared to monolithic zirconia, though bone resorption was lower. Corado et al. [37] provided a critical insight, demonstrating that traditional lithium disilicate glass-ceramics exhibited higher flexural strength than ZLS materials. They noted that the addition of zirconia can decrease overall crystallinity and potentially introduce microcracks, slightly reducing flexural strength and hardness [37,58,59]. This highlights a potential trade-off, where the incorporation of zirconia for strength reinforcement might, in some formulations, slightly compromise the maximum achievable mechanical properties of the glass-ceramic matrix.

Limitations and future directions

This review acknowledges several limitations within the primary literature. A primary constraint of the included in-vitro studies, such as Ko et al. [34], is the reliance on static loading, which does not fully replicate the complex, dynamic stresses of the oral environment. As such, these studies universally recommend long-term clinical trials to validate their laboratory findings. The clinical studies included [18,28,33], while promising, were limited by their relatively short follow-up periods, small sample sizes, and non-randomized designs. These investigators explicitly called for future research with larger cohorts, longer observation times, and randomized controlled methodologies to draw more robust clinical conclusions [18,28,33]. For the clinician, this underscores the necessity of a careful, case-specific selection process, weighing factors such as flexural strength, translucency, fracture toughness, and biocompatibility to achieve an optimal and satisfactory patient outcome [60,61].

## Conclusions

The current body of evidence largely supports the use of a zirconia coping veneered with lithium disilicate as a viable and aesthetically promising treatment modality. The bilayered approach leverages the high strength of zirconia and the superior aesthetics of lithium disilicate, demonstrating favorable mechanical performance and clinical outcomes in the short to medium term. However, the promising results from in-vitro studies require confirmation through well-designed, long-term clinical trials. Future research should prioritize prospective, randomized clinical studies with larger sample sizes and extended follow-up periods to definitively establish the clinical longevity, aesthetic stability, and periodontal response associated with these restorations.
